# Supplementary figures and images for: Defining Pseudo-Haplotype Analysis Reveals Multi-Gene Genetic Pattern Across BAF Chromatin Remodeling Complexes
Source: bioRxiv. 2026 Jun 26:2026.06.22.732952. Preprint. [Version 1] doi: 10.64898/2026.06.22.732952 (PMC13320759; doi:10.64898/2026.06.22.732952)

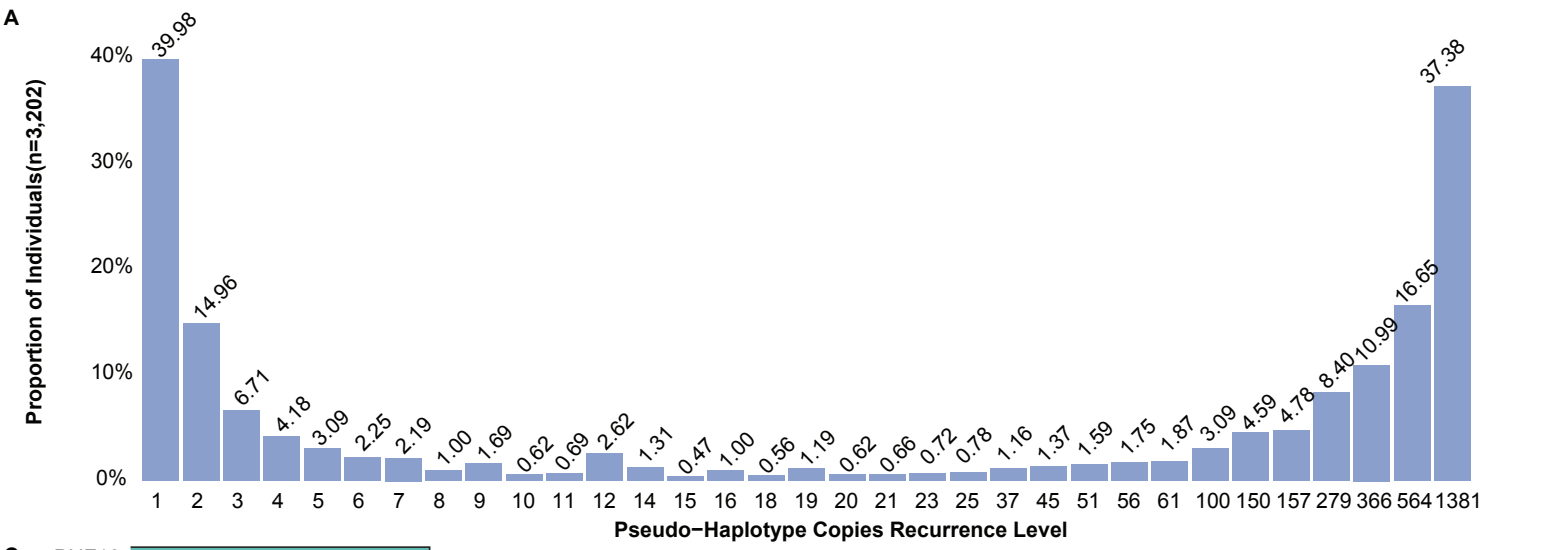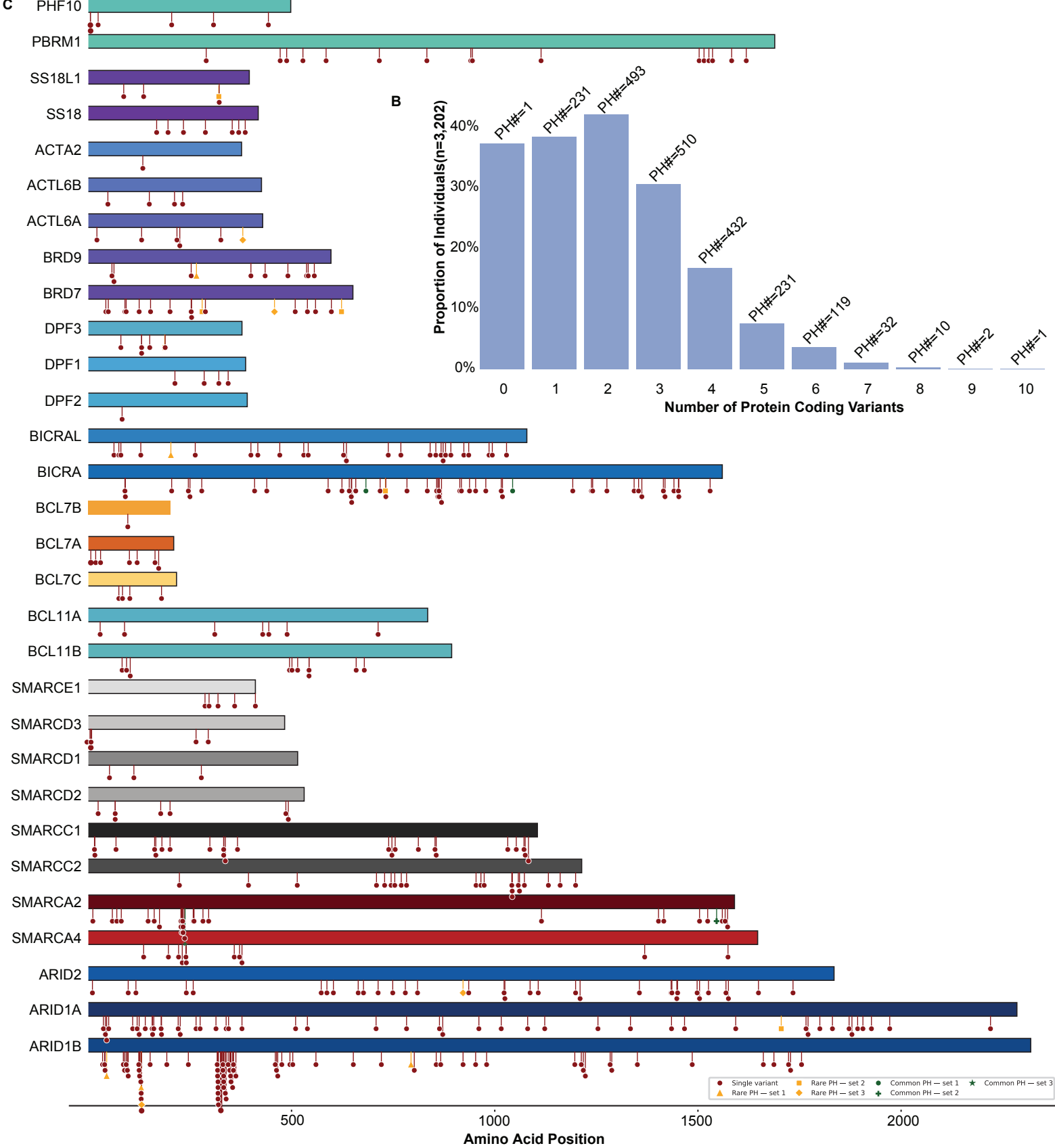

Supplement: Supplement 1 [file media-1.pdf]
